# Exploring the genetic background of the botulism neurotoxin BoNT/B2 in Spain

**DOI:** 10.1128/spectrum.02380-23

**Published:** 2023-09-26

**Authors:** Sylvia Valdezate, Gema Carrasco, María J. Medina, Noelia Garrido, Silvia del Pino, Monica Valiente, María P. Pallarés, Pilar Villalon

**Affiliations:** 1 Reference and Research Laboratory for Taxonomy, National Centre of Microbiology, Instituto de Salud Carlos III, Majadahonda, Madrid, Spain; 2 Veterinary Unit, Animal Department, Instituto de Salud Carlos III, Majadahonda, Madrid, Spain; Tainan Hospital, Ministry of Health and Welfare, , Tainan, Taiwan

**Keywords:** *Clostridium botulinum*, botulism, food-borne, infant, neurotoxin, BoNT, BoNT/B2, *bont/b2*, flaVR, ST, Spain

## Abstract

To determine whether the neurotoxin BoNT/B2 causing botulism in Spain is clonal, the genetic diversity and phylogenetic relationships of *Clostridium botulinum* from food-borne episodes and infant cases of the condition were explored. The botulinum toxin gene (*bont*) subtype, the variable region of the flagellin gene (*fla*VR), and a seven-gene multi-locus sequence type were examined by sequencing 37 BoNT-positive cultures obtained over the period 2010 to 2022. Out of 37 botulism events, 16 food-borne episodes and 16 infant cases were associated with *bont/b*2. Eight *bont/b*2 alleles were detected [nucleotide distance range 0.0259–0.415%, Hunter and Gaston discrimination index (HGDI) 0.71]. The most common *bont/b*2 allele corresponded to that of strain Prevot 25 NCASE and its single and double locus variations (87.5%). Four known *fla*VR types were identified (HGDI 0.79), along with one previously unknown (*fla*VR-15). Sixteen sequence types (STs) (HGDI 0.89) were recorded including seven new STs (ST164–ST170; 10 new alleles) and five new STs (ST171–ST175; with new allele combinations) were also noted. Correlations among some STs and *fla*VR types were seen. Overall, the present results show that the combined analysis of *bont/b*2-*fla*VR-ST at the nucleotide level could be used to track botulism events in Spain. The neurotoxin BoNT/B2 has largely been responsible for human botulism in Spain. The polymorphism analysis of *bont/b2*, *fla*VR typing, and sequence type determinations, revealed a wide variety of clones to be responsible for human botulism, ruling out a common source of acquisition.

**IMPORTANCE:**

Botulism, a potentially fatal disease, is classically characterized by a symmetrical descending flaccid paralysis, which if left untreated can lead to respiratory failure and death. Botulinum neurotoxin (BoNT), produced by certain species of Clostridium, is the most potent biological toxin known, and the direct cause of botulism. This study characterizes the acquisition in Spain of two forms of botulism, i.e., food-borne and infant botulism, which are largely caused by the main neurotoxin BoNT/B2. Polymorphism analysis of the bont/b2 gene, typing of the flagellin variable region sequence (flaVR), and multilocus sequence typing, were used to explore the genetic background of *Clostridium botulinum* group I. To our knowledge, this is the first phylogenetic and typing study of botulism undertaken in Spain.

## INTRODUCTION

Botulism remains a public health concern. Botulism neurotoxins (BoNTs) are listed as Category A (highest risk) agents by the U.S. Centers for Disease Control and Prevention (http://emergency.cdc.gov/bioterrorism). BoNTs irreversibly block acetylcholine release at neuromuscular synapses, resulting in motor and autonomic muscular blockade manifesting as progressive, symmetrical, descending flaccid paralysis, and even fatal asphyxia through diaphragm arrest (1 – 3). For a person with a body weight of 70 kg, the ingestion of just 70 µg of BoNT/A toxin is lethal. Death would also ensue if some 0.7–0.9 µg were inhaled or 0.09–0.15 µg were intravenously administered (4).

BoNTs—zinc metalloproteases that share the same structure—have been classified into nine serotypes depending on their antigenic activity (BoNT/A to BoNT/G, H or F/A, X), and further subdivided into more than 41 subtypes according to their sequence variations (subtypes A1–A8, B1–B8, E1–E12, and F1–F8) (2, 3).

BoNTs are produced by seven clostridial species: proteolytic *Clostridium botulinum* group I or *Clostridium parabotulinum*, non-proteolytic *C. botulinum* group II organisms, BoNT/C- and BoNT/D-producing bacteria included in the newly proposed species “*Clostridium novyi sensu lato,*” and *Clostridium argentinense*, *Clostridium baratii*, *Clostridium butyricum*, and *Clostridium sporogenes* (5). Proteolytic *C. botulinum* group I forms spores strongly resistant to heat and is primarily responsible for human botulism (2). The BoNT/C, /D, /C/D, and /D/C types mostly affect domestic and wild birds and cattle, but have zoonotic potential (6). The presence of *bont*-related sequences in non-clostridial strains ha been detected in the genomes of the *Weisenella oryzae* (*bont*/Wo or *bont*/I), *Enterococcus faecalis* (*bont*/J or *ebont*/F or *bont*/En), of *Chryseobacterium piperi* (*Cp*1), *Paraclostridium bifermentans* (the paraclostridial mosquitocidal protein 1, PMP1), but no consensus has been reached on their validity (3, 7).

The BoNT types actually produced are strain-independent. Most strains produce only one type, but others are bivalent [Ab, Ba, Af, Bf (the lowercase letter denoting lesser production)] or trivalent (e.g., strain Af84, which produces BoNT/A2, F4, and F5), and yet others possess silent neurotoxin genes (5, 8, 9). All *bont* genes are located within a neurotoxin cluster, either on the chromosome, on plasmids, or on phages, depending on the species and the bacterial strain. This supports the idea that horizontal *bont* transfer occurs between *Clostridium* and non-*Clostridium* strains.

The neurotoxin cluster arranges into one of two conformations, one containing hemagglutinin (HA) genes (*ha*17, *ha*33, and *ha*70), and the other *orf*X genes which are of unknown function (*orf*X1, *orf*X2, and *orf*X3) (2, 3, 8 – 11).

Human botulism, mainly associated with groups I and II, occurs in three main forms: food-borne botulism, botulism by intestinal colonization (including infant botulism), and wound botulism. Food-borne botulism occurs after the ingestion of pre-formed BoNT in food, while infant botulism is caused by spores germinating in the colon, leading to BoNTs being produced *in situ*. The ability to form endospores is critical to clostridial pathogenicity.

The active neurotoxin consists of a light chain (L, about 50 kDa) and a heavy chain (H, about 100 kDa), which remain linked by a disulfide bridge. Three distinct domains: L-chain containing α-helices, and β-strands and including the catalytic zinc-binding protease motif (His-Glu-XX-His); the N-terminal part of the H-chain (HN) forming two unusually long and twisted α-helices involved in the translocation of the L-chain into the cytosol; and the C-terminal part of the H-chain (HC) consisting of two distinct subdomains (H_CN_ and H_CC_). Mainly through the Hcc subdomain (the most divergent), the different BoNTs types/subtypes interact with distinct membrane receptors on demyelinated terminal nerve endings. The L-chain cleaves different intracellular SNARE proteins (soluble N-ethylmaleimide-sensitive factor attachment protein receptor) and form a complex that is essential for synaptic vesicle exocytosis in neurons: SNAP-25 (synaptosomal-associated protein 25 , the target for BoNT/A, C1, and E), syntaxin 1 (the target for BoNT/C1), and VAMP1/2/3 (vesicle-associated membrane protein, the target for BoNT/B, D, F, and G). The cleavage of the SNARE proteins blocks neurotransmission and causes flaccid muscle paralysis. After the disease develops, its treatment is problematic and mortality is high. Treatment includes trivalent or heptavalent equine botulinum antitoxin plus intensive care support, but even available between 5% and 10% of patients with food-borne botulism die (1, 3, 12, 13).

## MATERIALS AND METHODS

### Patients and samples

From 2010 to 2022, samples from 268 patients with suspected botulism or related neurological disorders (range 11–41 cases/year; mean 19.5) were studied at the National Centre of Microbiology of the Institute of Health Carlos III (Madrid, Spain). Seventy patients had laboratory-confirmed botulism (patients with at least one positive diagnostic test, range 1–13 cases/year; mean 4.4 per year), 34 food-borne and 36 infant botulism, as revealed by positive standard mouse bioassay (SMB, authorized by the corresponding institutional animal care) involving serum, stools, or gastric fluid, and/or by multiplex PCR of *bont* genes in stool cultures (14 – 17). Forty-eight patients were SMB-positive, while PCR confirmed botulism in 22 patients (31.43%) with SMB-negative results (19 food-borne and 3 infant cases). Thirty-seven positive cultures out of 70 patients [15 SMB(+)/PCR(+) and 22SMB(−)/PCR(+)] could be BoNT subtyped (10).

### Molecular surveillance by *bont/b2*, *fla*VR, and multi-locus sequence type analysis

To determine the alleles (variant forms of a gene) of the main subtype involved in Spain, *bont/b2* (3,876 nucleotides, 1,292 amino acids) was analyzed (10), and blastn/blastp comparisons made with available sequences in the GenBank database. Maximum likelihood (ML) phylogenies of the *bont/b2* gene, with the analysis of 62 sequences including those from other locations and representative of BoNT/B serotypes, were conducted using MEGA7 software.

To assess genetic relatedness, cultures and strains were typed by sequencing the variable region of flagellin A (*fla*VR) (18), and by multi-locus sequence typing with a seven-loci scheme (*aro*E, *mdh*, *ace*K, *opp*, *rpo*B, *rec*A, and *hsp*60) for the elucidation of evolutionary lineages (19). Allelic numbers and sequence types (STs) were identified by querying the C. *botulinum* multi-locus sequence type (MLST) PubMLST database (https://pubmlst.org/organisms/clostridium-botulinum) (20). New alleles and new STs were submitted to this database. The genetic relationships among the STs of *C. botulinum* BoNT/B2 within Spain, and with respect to those BoNT/B found in other countries were conducted using the goe-BURST routine (provided with PHYLOVIZ 2.0 software: https://online.phyloviz.net/index).

## RESULTS

### Predominance of botulism subtype BoNT/B2

**Fig 1:**
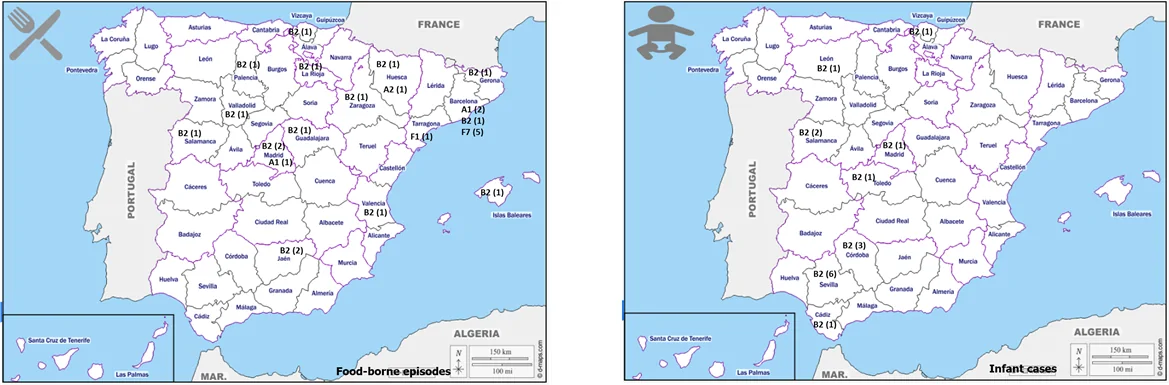
Distribution of BoNT subtypes for 21 food-borne episodes (on the left) and 16 infant cases (on the right) of laboratory-confirmed botulism in Spain over the period 2010–2022. Number of episodes and cases are included between parentheses. The maps were obtained from https://d-maps.com/carte.php?num_car=2208&lang=es.

BoNT subtyping was performed with 37 positive cultures (10); Fig. 1 shows the geographic distribution of these subtypes. Twenty-one food-borne episodes (involving one to five people) were caused by *C. botulinum* A1 (2 episodes), A2 (1 episode), B2 (16 episodes), F1 (1 episode), or *Clostridium baratti* F7 (1 episode). Sixteen recorded cases of infant botulism all involved BoNT/B2. All cases (food-borne and infant) involving this neurotoxin were caused by proteolytic *C. botulinum* group I (positive for the phenyllactate dehydratase subunit B gene, *fld*B, associated with phenylalanine metabolism in proteolytic clostridia) (21). Table 1 shows the epidemiological characteristics of the laboratory-confirmed botulism cases caused by BoNT/B2.

**TABLE 1:** Epidemiological and microbiological characteristics of laboratory confirmed infant and food-borne botulism cases in Spain (2010–2022)

Year	ID.^*a*^	Type (no. involved patients)*^b^*	Geographical location	*bont/b2* alelle*^c^* *^d^* (accession no.)	flaVR type^*e*^	ST^*f* *g*^	Alleles of the 7-loci MLST scheme^*g*^						
							*aroE*	*mdh*	*aceK*	*oppB*	*rpoB*	*recA*	*hsp*
2010	Bo7	Infant	Cordoba	Prevot 25 NCASE	15	58	13	8	15	9	8	7	8
2011	Bo12	Food-borne	Madrid	Prevot 25 NCASE	13	164	15	8	40	13	10	36	12
2011	Bo17	Infant	Salamanca	111	13	165	51	50	55	13	10	11	9
2011	Bo32	Food-borne	Guadalajara	Prevot 25 NCASE	15	58	13	8	15	9	8	7	8
2012	Bo43	Food-borne	La Rioja	Prevot 25 NCASE	13	80	15	8	40	13	10	11	12
2012	Bo47	Infant	Vizcaya	111	15	166	15	8	56	9	31	11	34
2013	Bo65	Food-borne (^*b*^)	Salamanca	SLV-1154I (OQ683359)	1	53	11	8	11	9	8	6	8
2014	Bo76	Infant	Seville	Prevot 25 NCASE	4	167	13	1	57	9	7	6	8
2014	Bo81	Food-borne (^*b*^)	Palencia	Prevot 25 NCASE	13	164	15	8	40	13	10	36	12
2015	Bo95	Food-borne	Jaen	SLV-1030D (OQ683360)	4	171	13	8	28	9	8	7	8
2016	Bo110	Food-borne	Vizcaya	SLV-1030D (OQ683360)	15	58	13	8	15	9	8	7	8
2016	Bo114	Infant	Seville	SLV-1030D (OQ683360)	na^*h*^	na^*h*^	na	na	na	9	na	na	na
2016	Bo119	Infant	Cadiz	Prevot 25 NCASE	4	168	13	5	57	9	7	6	8
2017	Bo122	Infant	Madrid	Prevot 25 NCASE	15	58	13	8	15	9	8	7	8
2017	Bo130	Infant	Seville	Prevot 25 NCASE	15	58	13	8	15	9	8	7	8
2017	Bo133	Food-borne (^*b*^)	Mallorca	Prevot 25 NCASE	4	172	13	8	15	9	7	7	8
2018	Bo147	Infant	Salamanca	111	na	na	13	na	34	26	19	18	14
2018	Bo148	Food-borne	Zaragoza	SLV-1030D (OQ683360)	4	52	13	5	28	9	8	7	8
2019	Bo159	Food-borne (^*b*^)	Huesca	SLV-_279A_ (OQ683361)	15	173	13	5	15	9	8	7	8
2019	Bo161	Food-borne (^*b*^)	Madrid	Prevot 25 NCASE	1	53	11	8	11	9	8	6	8
2019	Bo163	Food-borne	Jaen	Prevot 25 NCASE	4	168	13	5	57	9	7	6	8
2019	Bo164	Infant	Seville	SLV-1030D (OQ683360)	4	174	15	5	28	9	16	7	8
2019	Bo191	Infant	Cordoba	Prevot 25 NCASE	4	169	13	8	57	9	7	6	8
2019	Bo196	Infant	Seville	SLV-1127F (OQ683362)	15	58	13	8	15	9	8	7	8
2020	Bo197	Infant	Leon	111	na	175	9	18	24	12	10	7	8
2020	Bo209	Food-borne (^*b*^)	Barcelona	DLV-_3828G, 3843A_ (OQ683363)	1	53	11	8	11	9	8	6	8
2020	Bo216	Food-borne	Valladolid	Prevot 25 NCASE	13	164	15	8	40	13	10	36	12
2021	Bo220	Food-borne (^*c*^)	Valencia	Prevot 25 NCASE	1	53	11	8	11	9	8	6	8
2021	Bo225	Infant	Seville	SLV-1030D (OQ683360)	4	52	13	5	28	9	8	7	8
2021	Bo236	Infant	Toledo	Prevot 25 NCASE	15	58	13	8	15	9	8	7	8
2022	Bo264	Infant	Cordoba	Prevot 25 NCASE	15	58	13	8	15	9	8	7	8
2022	Bo267	Food-borne	Gerona	MLV (OQ683364)	na	170	52	47	58	13	10	11	8
Diversity index (HGDI)^*h*^				0.71	0.79	0.89	0.65	0.58	0.86	0.36	0.69	0.72	

^*a*^ ID^1^—identification number.

^*b*^ no. involved patients— number of patients with botulism in the same food-borne episode (not indicated when only one patient was affected).

^*c*^ *bont/b2* allele—the alleles are defined in terms of their identity with respect to the corresponding sequence of *C. botulinum* strain 111 and strain Prevot 25 NCASE (GenBank accession nos. BAC22064 and EF033129.1, respectively).

^*d*^ SLV, DLV, and MLV—single, double and multi locus variants with respect to the sequence of strain Prevot 25 NCASE, with positional and amino acid changes shown [nucleotide change in subscript] (GenBank accession nos. OQ683359-OQ683364). For the MLV allele, amino acid and silent nucleotide changes are indicated in the text.

^*e*^ The flaVR type is coded as described by Paul et al. (18) and Woudstra et al. (22). The new flaVR-15 for *C. botulinum* CDC 53174 is designated CP013247.1.

^*f*^ ST—sequence type or allelic profile (MLST scheme) in pubMLST (https://pubmlst.org/organisms/clostridium-botulinum).

^*g*^ na—not available (no results for PCR or sequencing).

^*h*^ HGDI—Hunter-Gaston discriminatory index.

### Distribution of *bont/b2* alleles

Eight *bont/b2* alleles were detected. An allele identical to *bont/B2* of *C. botulinum* strain 111 (sequence accession BAC22064), the representative strain of BoNT/B2, was seen in four infant cases only. Another allele was identical to that of the strain Prevot 25 NCASE (sequence accession EF033129.1) (10); this was associated with nine food-borne episodes and eight infant cases. The *bont/b2* of the latter strain shows a difference of just 0.15% (two amino acids and six nucleotides) with respect to strain 111. Table 1 shows the distribution of the different *bont/b2* alleles.

**Fig 2:**
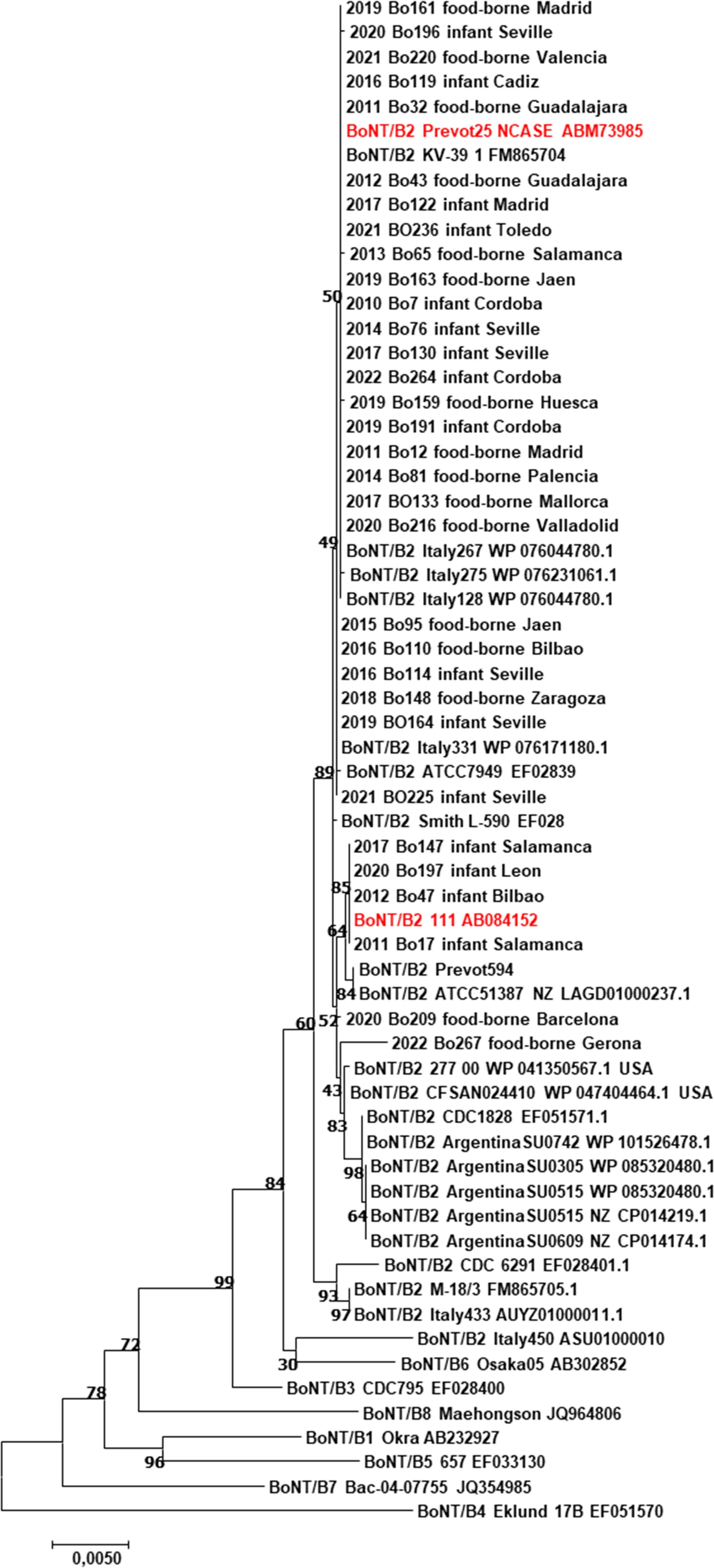
Maximum likelihood (ML) phylogenies of the Spanish *bont/B2* gene (coded as year case no. food-borne or infant province), with the analysis of 62 sequences including those from other locations and representative of BoNT/B serotypes. The reliability of the ML topologies was assessed via 1,000 bootstrap replications. The percentage of trees in which the associated taxa clustered together is shown next to the branches. The tree is drawn to scale, with branch lengths measured in terms of the number of substitutions per site. Evolutionary analyses were conducted using MEGA7 software.

Six new single locus variants (SLVs, sequence types that differ by one allele type) and double variants (DLVs, sequence types that differ by two allele types) with respect to bont/B2 of the Prevot 25 NCASE strain were identified: (i) SLV-1154I (amino acid change); (ii) SLV-1030D (six cases); (iii) SLV-_279A_ (silent nucleotide substitution); (iv) SLV-1127F; (v) DLV-_3828G/3843-A_; and (vi) a MLV with six amino acid (579R-820G-871K-884K-889N-1030D) and seven silent nucleotide changes (1677C-1788G-1809C-2067G-2665A-2676A-3822T), showing 0.46% difference to the BoNT/B2 (in protein) of strain 111 (new subtype > 2.6%) (3). Fig. 2 shows the relationships among the Spanish *bont/b2* alleles (nucleotide distance range 0.0259–0.415%), and with others previously described in other countries (8, 23).

### *fla*VR and STs of Spanish *bont/b2*

Three described *fla*VR-types*—fla*VR-1, *fla*VR-4, and *fla*VR-13 (18, 22)—were detected in four, nine, and five samples, respectively (Table 1). A fourth one, an undescribed *fla*VR-type (86.04% identity with *fla*VR-1), found to be identical to a sequence for *C. botulinum* BoNT/A strain CDC 53174 and strain Mauritius (sequence accession CP013247.1 and CP013849.1), was identified in 10 of the received samples. It was codified as *fla*VR-15.

**Fig 3:**
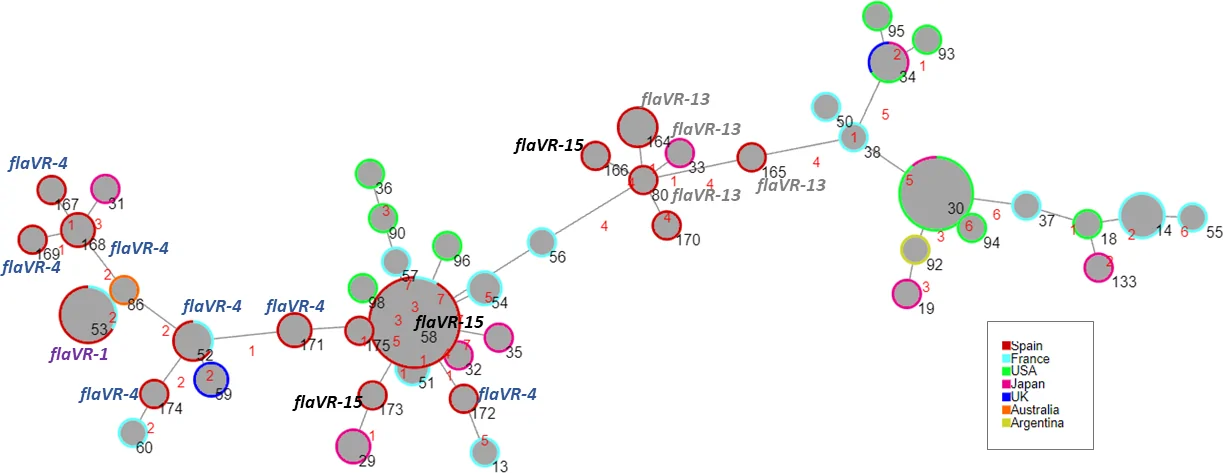
Genetic relationships among the STs of *C. botulinum* BoNT/B within Spain, and with respect to those found in other countries. The goe-BURST routine (provided with PHYLOVIZ 2.0 software: https://online.phyloviz.net/index), and the criterion of seven shared alleles, were used to distinguish clonal complexes among 83 isolates. Forty-seven STs for BoNT/B were available in PubMLST (https://pubmlst.org/organisms/clostridium-botulinum). Black and red numbers indicate STs and genetic distances. The *fla*VR types for the received Spanish samples are included.

Sixteen STs were revealed by PubMLST database queries (20). Four had been previously described in France (a neighboring country of Spain) as ST52, ST53, ST58, and ST80, with two, four, eight, and one cases, respectively. Seven new STs (ST164-170) with 10 new alleles (*ace*K-55–56-57-58, *aro*E-51-52, *mdh*-50, *rpo*B-31, *rec*A-36, and *hsp*-34), and five new STs (ST171-175) with new allelic combinations [detected in one sample each, except for ST164 and ST168 (in three and two samples)] were identified for the first time (Table 1 and Fig. 3). Via BURST analysis of the PubMLST data, the current 182 *C*. *botulinum/C. sporogenes* STs were gathered into 19 groups and 32 singletons (20). The STs found in Spain were gathered into group 2 (ST52, ST53, ST58, ST167-169, and ST171-174), group 5 (ST175), and group 6 (ST80, ST164), with three singletons (ST165, ST166, and ST170) (Fig. S1). Correlations among STs and *fla*VR-types were detected for ST53 and *fla*VR-1, ST52 and ST168 and *fla*VR-4, ST164 and *fla*VR-13, and ST58 and *fla*VR-15 (Fig. 3).

## DISCUSSION

In the European Union, botulism case numbers have remained stable at ≈100 cases/year with an overall notification rate of 0.02 cases/100,000 inhabitants (as occurs in Spain) (1, 24), although the botulism form (food-borne or infant) and incidence vary according to the reporting countries. The highest incidences have been recorded in Italy and Romania. Food-borne botulism outbreaks are, however, reported worldwide, and depend notably on dietary habits and culinary traditions (1, 25). An unusually high prevalence of infant botulism is reported in the United States (100 cases per year), especially northern California, eastern Pennsylvania, southern New Jersey, and northern Delaware. The same has been described for Argentina (Buenos Aires and Mendoza) and Spain (23, 26). A higher incidence of infant cases in Andalusia (10/16), especially Seville, with no clear association with any particular food or environmental source or risk factor or sex (nine males and seven females). This form of the disease only occurs in infants (typically when they are between 1 and 6  months old, but sometimes up to 12  months of age) given their immature gut physiology and/or underdeveloped gut microbiota. The source of the spores in affected infants remains unknown. They may come from contaminated soil and dust from nearby construction sites, via the consumption of powdered milk, natural sweeteners, corn syrup, or medicinal herbs. The real impact of infant botulism is likely underestimated worldwide given diagnostic difficulties and the condition’s non-specific presentation (12, 13). At the time of the present study, iatrogenic botulism caused by the therapeutic use of BoNT (for the treatment of spasticity, focal dystonia, hemifacial spasm, hyperhydrosis, strabismus, chronic migraine, and cosmetic) was unreported.

In Spain, the subtype BoNT/B2 was by far the most frequently encountered neurotoxin. The predominance of specific BoNT types/subtypes in different geographical areas had already been noted, with BoNT/B involved in ≈90% of cases from the European Union, BoNT/A1 and BoNT/B1 predominant in the United States, and BoNT/B2 predominant in Argentina (1, 23). Moreover, the present findings show strong predominance (87.5%) of the Prevot 25 NCASE *bont/b2* allele and its variations (SLV and DLV) in Spain. Analysis of the nucleotide differences of the *bont/b2* gene [Hunter and Gaston discrimination index (HGDI) 0.71] may be of help as a first step in botulism surveillance. Strong *bont/b2* identity with the Prevot 25 NCASE strain has been also reported for Italian strains (8). It should be noted that BoNT/B has the greatest degree of inter-subtype and intra-subtype variability of all BoNTs (0.8–1.9%), perhaps as the consequence of more frequent recombination events allowing the subtype’s proliferation (2).

Two studies, based on the sequence of the variable region of *fla*A revealed the genetic diversity of the flagellin genes of the *C. botulinum* group I and II strains, affording a simple genotyping method (18, 22). Different strains with the same or no BoNT can share the same *fla*VR type. Seventy-five percent of the European strains carried four types (*fla*VR-1, *fla*VR-3, *fla*VR-4, and *fla*VR-5), and their distribution may vary with geographical origin (22). In Spain, two *fla*VR types out of four, *fla*VR-4 and *fla*VR-15, were the most frequent (each one accounting for nearly one third of all cases). No correlation with geographical origin could be confirmed.

MLST has been used for genetic diversity and phylogenetic analyses of *C. botulinum*, but with varying success. It has been reported an efficient method for strain discrimination and for phylogenetic analyses of *C. botulinum* BoNT/A2 (19) and BoNT/B2 (27). However, in other studies involving *C. botulinum* BoNT/A2, BoNT/A3 and BoNT/B5, and BoNT/A(B) it has not given such good results, probably due to the limited genetic diversity of these latter strains (28 – 30). Sixteen STs were detected, 12 of which were new (with 10 new alleles). ST58, the most common ST, was detected mainly in cases of infant botulism (six out of eight cases). No other known ST for *bont/b2* described in other countries [i.e., ST19, ST29, ST31-33, ST37, ST38, ST51, ST54-57, ST59, and ST92 (20)] was identified. In the present study, the seven-loci scheme returned a sufficiently good diversity coefficient (HGDI 0.89), and is therefore useful for the molecular typing and phylogenetic characterization of Spanish *C. botulinum* subtype BoNT/B2.

Discrepancies between bont/b genes and chromosome clonal phylogeny have been described, probably because of these genes' considerable lateral spread by recombination among different lineages leading to wide heterogeneity in *C. botulinum* group I populations (2, 5, 8, 10, 23, 27). Pangenomic analysis of the *C. botulinum* group I strains, which have relatively stable genomic components, is an almost perfect way of studying genetic population structures and botulism events (8, 9, 23, 27 – 30). In its absence, however, the combined analysis of *bont/b*2-*fla*VR-ST at the nucleotide level appears to offer a suitable way of tracking botulism events in Spain. Despite the predominance of the Prevot 25 NCASE strain *bont/b*2 allele and its SLVs and DLVs, the four *fla*VR-types and the 16 STs revealed different evolutionary lineages to be responsible for human botulism in our country. In France, *C. botulinum* BoNT/A (the main neurotoxin in this country), and BoNT/B also showed a wide genetic diversity, probably due to multiple and independent genetic rearrangements (27), but not to a single evolutionary lineage as could have happened in other countries (28 – 30). High heterogeneity of the circulating strains is also observed via multiple-locus variable number of tandem repeat analysis during botulism surveillance activities in Italy, other Mediterranean countries, without detection of any regional or temporal clustering (31).

In this scenario of diversity, the behavior of the *fla*VR and *ace*K genes in typing (HGDI ≥ 0.8, Table 1) indicated their potential use as first-line markers in the epidemiological surveillance of botulism via BoNT/B2 in Spain. Despite the temporal and geographical relationships between the examined disease events, no clustering or any further epidemiological link was detected.

In conclusion, the BoNT/B2 subtype is mainly responsible for food-borne episodes of botulism in Spain, and, for now, is the only one involved in infant cases. Polymorphism analysis of *bont/b2*, *fla*VR typing, and sequence type determination, showed, for first time in Spain, that different clones are responsible for human botulism. The genetic background of the neurotoxin BoNT/B2 has been explored with success to track botulism events in Spain.

## Data Availability

New *bont/b2* alelles were assigned the GenBank accession nos. OQ683359-OQ683364.

## SUPPLEMENTAL MATERIAL

Fig. S1 spectrum.02380-23-s0001.tif BURST groups for the currently known 182 STs.

ASM does not own the copyrights to Supplemental Material that may be linked to, or accessed through, an article. The authors have granted ASM a non-exclusive, world-wide license to publish the Supplemental Material files. Please contact the corresponding author directly for reuse.
