# Supplementary figures and images for: Exploring the genetic background of the botulism neurotoxin BoNT/B2 in Spain
Source: Microbiol Spectr. 2023 Sep 26;11(5):e02380-23. doi: 10.1128/spectrum.02380-23 (PMC10581064; doi:10.1128/spectrum.02380-23)

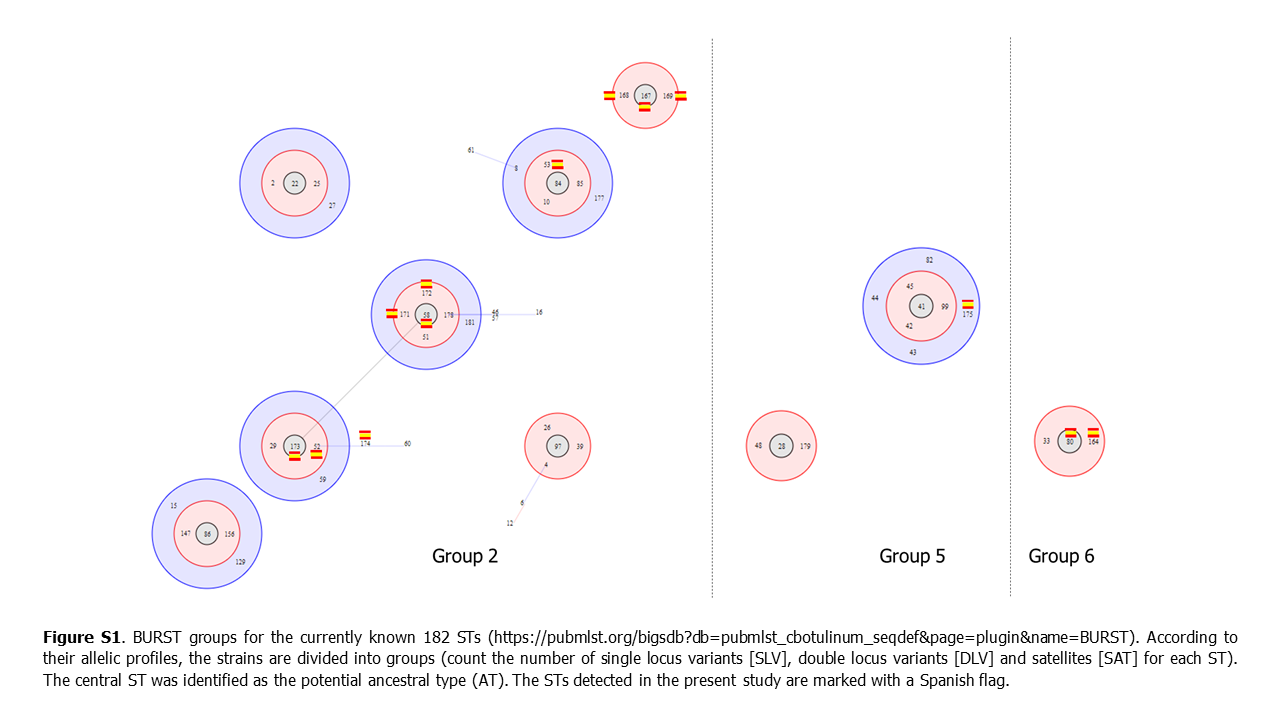

Supplement: Fig. S1 — BURST groups for the currently known 182 STs. [file spectrum.02380-23-s0001.tif]
